# Supplementary material for: Odor classification: Exploring feature performance and imbalanced data learning techniques
Source: PLoS One. 2025 May 28;20(5):e0322514. doi: 10.1371/journal.pone.0322514 (PMC12118925; doi:10.1371/journal.pone.0322514)
Supplement: Supplementary File 2 — (PDF) [file pone.0322514.s002.pdf]

# Odor Classification: Exploring Feature Performance and Imbalanced Data Learning Techniques **(Supplementary Material)**

Durgesh Ameta[1,2◕], Surendra Kumar[3¤], Rishav Mishra[3¤], Laxmidhar Behera[1,5¶], Aniruddha Chakraborty[4¶], Tushar Sandhan[5‡]

**1** Indian Knowledge System and Mental Health Applications Centre, Indian Institute of Technology, Mandi, 175005, India
**2** Indian Knowledge System Centre, ISS, Delhi, 110065, India
**3** School of Electronics, Indian Institute of Information Technology, Una, 177209, India
**4** School of Basic Sciences, Indian Institute of Technology, Mandi, 175005, India
**5** Department of Electrical Engineering, Indian Institute of Technology, Kanpur, 208016, India

◕Author contributed to Conceptualization, Data collection, Analysis, and paper writing.
¤These authors contributed to Data collection, Analysis, and paper writing.
¶These authors contributed to Supervision, Interpretation, Review and editing.
‡Author contributed to Conceptualization, Supervision, Interpretation, Review and editing
* durgesgameta@gmail.com

## Architecture of models used for classification

As described in **MATERIALS AND METHODS** section of the manuscript, we provide more implementation details of models used for classification in this section. We utilized focal loss in the CSMLP model to deal with class imbalance [1–4]. For the activation function in CSMLP, we have used Relu, and at the final layer, we used sigmoid. The CSMLP Model was trained with Adam [5](keras-optimizer), and dropout was used as a regularizer to prevent overfitting. Along with CSMLP other models used were Random Forest (RF), Binary Relevance (BR), Classifier Chains (CC). Since BR and CC use RF as the base classifier, we have only discussed the hyperparameters of the RF.

### Random Forest classifier

We used the default hyperparameters for the Random Forest classifier model, which provided optimal results for our use case. The hyperparameter details for the RF are as follows:

| Hyperparameter | Value |
|---|---|
| n_estimators | 100 |
| min_samples_leaf | 1 |
| min_samples_split | 2 |
| max_features | Sqrt |
| criterion | Gini |

## CSMLP

Architectural details of the CSMLP used for classification are:

| Layers | Shape | Parameters |
|---|---|---|
| dense(Dense) | (None,800) | 171200 |
| dropout(Dropout) | (None,800) | 0 |
| dense_1(Dense) | (None,300) | 240300 |
| dropout_1(Dropout) | (None,300) | 0 |
| dense_2(Dense) | (None,150) | 45150 |
| dense_3(Dense) | (None,109) | 16459 |

Loss Function = Focal Loss
$\gamma$ =1.5,
$\epsilon = e^{-7}$,
epochs =100

'he-uniform' kernel initializer and ReLU activation were applied for all the dense layers except the output layer. For the output layer, a sigmoid activation function was used.

# Classification Results (Other Metrics)

| Algorithm | Dataset | Baseline | Multi-Label Random Under-Sampling (MLRUS) | | | Multi-Label Random Over-Sampling (MLROS) | | | % F1 Increase from Baseline |
|---|---|---|---|---|---|---|---|---|---|
| | | | 10 | 20 | 30 | 10 | 20 | 30 | |
| Random Forest (RF) | IGD_FP | 0.260<br>0.328<br>0.231<br>0.865<br>0.790 | 0.240<br>0.304<br>0.210<br>0.854<br>0.781 | 0.263<br>0.336<br>0.225<br>0.859<br>0.785 | 0.248<br>0.341<br>0.211<br>0.848<br>0.787 | 0.257<br>0.316<br>0.229<br>0.864<br>0.796 | **0.267**<br>0.330<br>0.237<br>0.861<br>0.785 | 0.265<br>0.314<br>0.237<br>0.860<br>0.792 | 2.692 |
| | Subset-IGD_FP | 0.207<br>0.297<br>0.190<br>0.860<br>0.758 | 0.211<br>0.387<br>0.195<br>0.870<br>0.774 | **0.223**<br>0.402<br>0.203<br>0.881<br>0.784 | 0.211<br>0.405<br>0.183<br>0.851<br>0.752 | 0.212<br>0.301<br>0.195<br>0.861<br>0.764 | 0.210<br>0.311<br>0.194<br>0.852<br>0.759 | 0.211<br>0.313<br>0.194<br>0.866<br>0.765 | 7.729 |
| | Subset-IGD_VS | 0.132<br>0.261<br>0.109<br>0.796<br>0.702 | 0.133<br>0.330<br>0.111<br>0.787<br>0.689 | 0.122<br>0.364<br>0.088<br>0.782<br>0.692 | 0.123<br>0.367<br>0.094<br>0.771<br>0.681 | 0.135<br>0.265<br>0.112<br>0.765<br>0.685 | **0.136**<br>0.265<br>0.113<br>0.778<br>0.697 | 0.135<br>0.265<br>0.112<br>0.689<br>0.677 | 2.941 |
| | Subset-IGD_MS | 0.173<br>0.326<br>0.148<br>0.787<br>0.729 | 0.159<br>0.355<br>0.129<br>0.815<br>0.724 | 0.130<br>0.264<br>0.105<br>0.803<br>0.710 | 0.152<br>0.393<br>0.117<br>0.810<br>0.708 | **0.176**<br>0.326<br>0.298<br>0.834<br>0.726 | 0.172<br>0.325<br>0.147<br>0.818<br>0.721 | 0.173<br>0.326<br>0.148<br>0.821<br>0.725 | 1.734 |
| Binary Relevance (BR) | IGD_FP | 0.284<br>0.304<br>0.285<br>0.865<br>0.793 | 0.263<br>0.285<br>0.256<br>0.857<br>0.789 | **0.290**<br>0.326<br>0.274<br>0.860<br>0.789 | 0.265<br>0.319<br>0.244<br>0.848<br>0.788 | 0.287<br>0.306<br>0.290<br>0.864<br>0.790 | 0.286<br>0.299<br>0.290<br>0.861<br>0.790 | 0.286<br>0.295<br>0.289<br>0.861<br>0.790 | 2.112 |
| | Subset-IGD_FP | 0.231<br>0.277<br>0.235<br>0.857<br>0.769 | 0.244<br>0.378<br>0.248<br>0.859<br>0.773 | **0.250**<br>0.380<br>0.239<br>0.862<br>0.793 | 0.233<br>0.352<br>0.228<br>0.861<br>0.775 | 0.237<br>0.285<br>0.240<br>0.860<br>0.773 | 0.237<br>0.287<br>0.239<br>0.859<br>0.767 | 0.239<br>0.286<br>0.242<br>0.855<br>0.767 | 8.225 |
| | Subset-IGD_VS | 0.146<br>0.246<br>0.125<br>0.830<br>0.725 | **0.171**<br>0.340<br>0.145<br>0.840<br>0.726 | 0.134<br>0.350<br>0.102<br>0.832<br>0.721 | 0.131<br>0.344<br>0.103<br>0.829<br>0.712 | 0.148<br>0.247<br>0.127<br>0.827<br>0.718 | 0.147<br>0.245<br>0.127<br>0.824<br>0.706 | 0.148<br>0.247<br>0.12<br>0.826<br>0.719 | 17.123 |
| | Subset-IGD_MS | 0.185<br>0.324<br>0.166<br>0.833<br>0.732 | 0.169<br>0.353<br>0.140<br>0.831<br>0.732 | 0.137<br>0.257<br>0.114<br>0.830<br>0.725 | 0.63<br>0.366<br>0.132<br>0.824<br>0.721 | **0.188**<br>0.326<br>0.168<br>0.835<br>0.736 | 0.187<br>0.325<br>0.167<br>0.831<br>0.743 | 0.186<br>0.326<br>0.166<br>0.833<br>0.736 | 1.621 |
| Classifier Chain (CC) | IGD_FP | 0.274<br>0.327<br>0.249<br>0.863<br>0.788 | 0.256<br>0.321<br>0.227<br>0.859<br>0.785 | **0.279**<br>0.353<br>0.241<br>0.863<br>0.796 | 0.258<br>0.338<br>0.226<br>0.851<br>0.789 | 0.270<br>0.325<br>0.245<br>0.862<br>0.788 | 0.272<br>0.326<br>0.248<br>0.861<br>0.788 | 0.272<br>0.317<br>0.248<br>0.862<br>0.791 | 1.824 |
| | Subset-IGD_FP | 0.216<br>0.318<br>0.203<br>0.863<br>0.779 | **0.239**<br>0.421<br>0.224<br>0.870<br>0.788 | 0.234<br>0.418<br>0.211<br>0.863<br>0.796 | 0.210<br>0.376<br>0.190<br>0.863<br>0.771 | 0.230<br>0.329<br>0.214<br>0.863<br>0.780 | 0.229<br>0.354<br>0.211<br>0.856<br>0.765 | 0.229<br>0.345<br>0.212<br>0.853<br>0.765 | 10.648 |
| | Subset-IGD_VS | 0.136<br>0.292<br>0.112<br>0.830<br>0.714 | **0.142**<br>0.365<br>0.117<br>0.830<br>0.714 | 0.127<br>0.368<br>0.093<br>0.832<br>0.708 | 0.127<br>0.358<br>0.098<br>0.819<br>0.700 | 0.138<br>0.294<br>0.113<br>0.827<br>0.731 | 0.138<br>0.293<br>0.114<br>0.825<br>0.716 | 0.137<br>0.292<br>0.113<br>0.823<br>0.712 | 4.411 |
| | Subset-IGD_MS | 0.176<br>0.349<br>0.149<br>0.834<br>0.745 | 0.165<br>0.377<br>0.132<br>0.833<br>0.737 | 0.128<br>0.285<br>0.103<br>0.825<br>0.722 | 0.161<br>0.392<br>0.125<br>0.819<br>0.712 | **0.179**<br>0.352<br>0.151<br>0.832<br>0.729 | 0.177<br>0.342<br>0.150<br>0.833<br>0.736 | 0.170<br>0.340<br>0.150<br>0.835<br>0.736 | 1.704 |
| Cost Sensitive MLP (CSMLP) | IGD_FP | 0.300<br>0.436<br>0.353<br>0.915<br>0.863 | 0.278<br>0.431<br>0.297<br>0.903<br>0.849 | 0.278<br>0.431<br>0.321<br>0.901<br>0.852 | 0.269<br>0.375<br>0.312<br>0.894<br>0.845 | **0.311**<br>0.368<br>0.372<br>0.915<br>0.861 | 0.310<br>0.377<br>0.347<br>0.915<br>0.861 | 0.301<br>0.344<br>0.348<br>0.913<br>0.859 | 3.666 |
| | Subset-IGD_FP | 0.229<br>0.627<br>0.248<br>0.899<br>0.825 | 0.231<br>0.584<br>0.274<br>0.904<br>0.841 | 0.224<br>0.547<br>0.290<br>0.895<br>0.830 | 0.206<br>0.534<br>0.250<br>0.891<br>0.806 | 0.233<br>0.485<br>0.275<br>0.898<br>0.831 | **0.253**<br>0.379<br>0.324<br>0.897<br>0.817 | 0.249<br>0.429<br>0.292<br>0.899<br>0.835 | 10.480 |
| | Subset-IGD_VS | 0.146<br>0.519<br>0.188<br>0.854<br>0.743 | **0.203**<br>0.433<br>0.230<br>0.873<br>0.806 | 0.154<br>0.554<br>0.182<br>0.860<br>0.762 | 0.131<br>0.543<br>0.183<br>0.843<br>0.479 | 0.156<br>0.411<br>0.195<br>0.855<br>0.750 | 0.163<br>0.351<br>0.210<br>0.855<br>0.750 | 0.167<br>0.286<br>0.232<br>0.855<br>0.750 | 39.041 |
| | Subset-IGD_MS | 0.118<br>0.614<br>0.137<br>0.852<br>0.753 | 0.112<br>0.605<br>0.145<br>0.851<br>0.762 | 0.104<br>0.588<br>0.136<br>0.843<br>0.743 | 0.108<br>0.663<br>0.128<br>0.828<br>0.704 | 0.131<br>0.556<br>0.153<br>0.851<br>0.754 | 0.139<br>0.455<br>0.159<br>0.849<br>0.749 | **0.147**<br>0.440<br>0.169<br>0.849<br>0.750 | 24.576 |

**Table 1.** This table compares the performance of RF, BR, CC, and CSMLP algorithms across various datasets and features. The evaluation uses Multi-Label Random Under-Sampling (MLRUS) and Multi-Label Random Over-Sampling (MLROS) techniques with sampling ratios of 10%, 20%, and 30%. Macro-averaged **F1-scores**, Precision, Recall and Micro-AUC-ROC, Macro-AUC-ROC are reported for each algorithm-dataset pair; The final column shows the percentage F1 score increase from the baseline to highlight improvements in handling class imbalance and classification accuracy.

## Feature Importance Analysis with SHapley Additive exPlanations (SHAP)

Explaining the classification models is crucial for developing an electric nose. This study used SHAP to determine feature importance in CSMLP for Subset-IGD_VS, Subset-IGD_MS, and Subset-IGD_FP. SHAP is a model-agnostic method that does not require model retraining, providing a fresh perspective on feature importance compared to traditional methods like permutation and the Gini index [6]. Hence, we present the SHAP Summary Plots. The CSMLP model described in the previous section was used for SHAP analysis for Subset-IGD_VS, Subset-IGD_MS and Subset-IGD_FP. This section presents SHAP summary plots for odors “Sweet” and “Green”.

### SHAP Summary Plots

Fig. 1, Fig. 2, Fig. 3 below are summary plots on top 20 best features for “Sweet” and “Green” classes for Subset-IGD_VS, Subset-IGD_MS, Subset-IGD_FP.

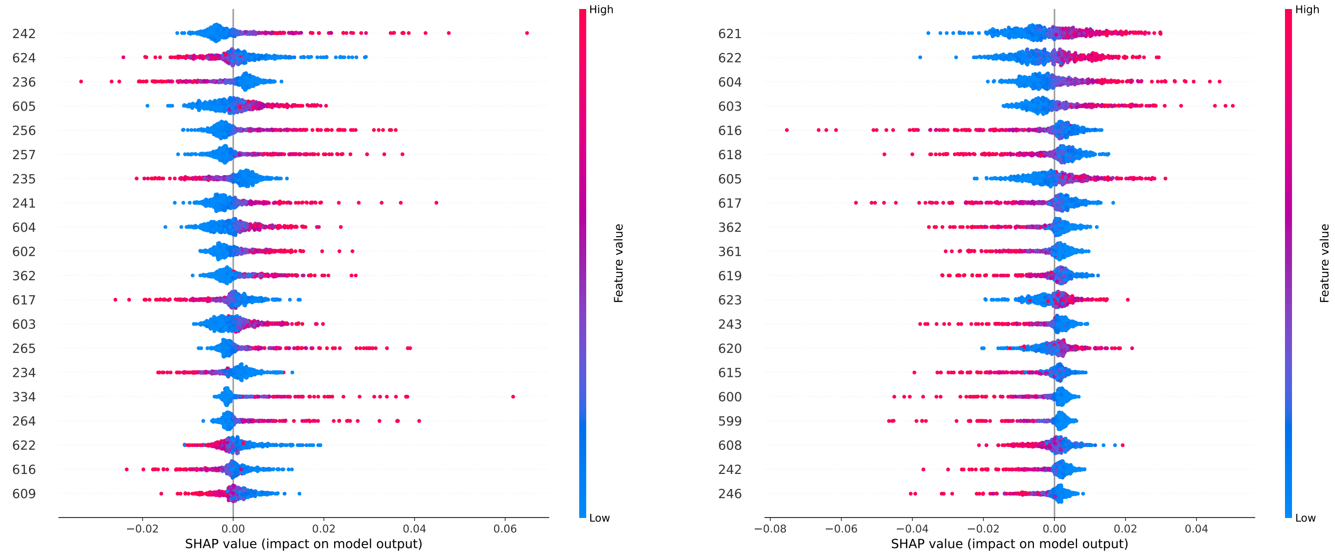


**(a)** Summary Plot for Subset-IGD_VS (Class: Sweet)

**(b)** Summary Plot for Subset-IGD_VS (Class: Green)

**Fig 1.** SHAP Summary plot between SHAP feature importance values vs 20 most important features illustrating SHAP feature importance values for Subset-IGD_VS on CSMLP model, focusing on “Sweet” and “Green” odor classes.

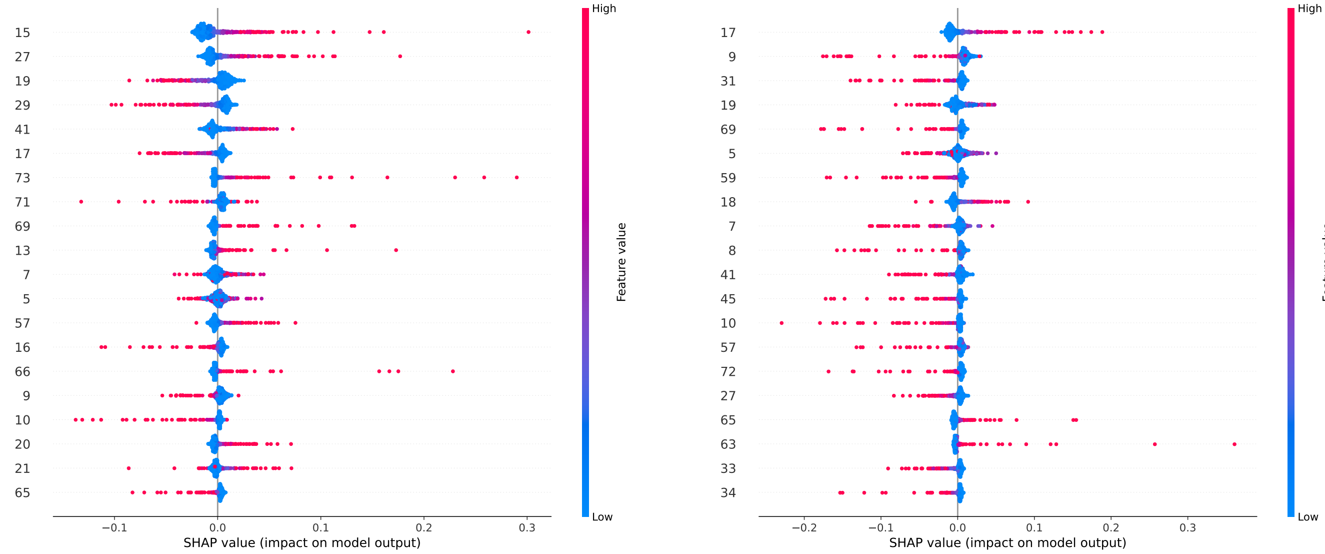


**(a)** Summary Plot for Subset-IGD_MS (Class: Sweet) **(b)** Summary Plot for Subset-IGD_MS (Class: Green)

**Fig 2.** SHAP Summary plot between SHAP feature importance values vs. 20 most important features illustrating SHAP feature importance values for Subset-IGD_MS on CSMLP model, focusing on "Sweet" and "Green" odor classes.

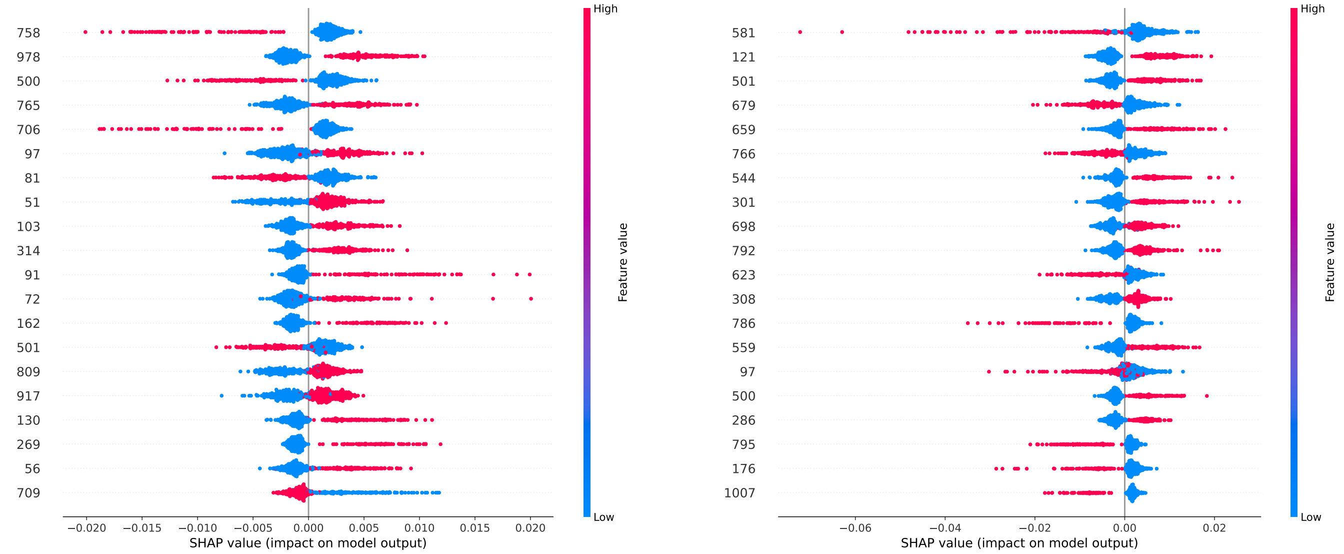


**(a)** Summary Plot for Subset-IGD_FP (Class: Sweet) **(b)** Summary Plot for Subset-IGD_FP (Class: Green)

**Fig 3.** SHAP Summary plot between SHAP feature importance values vs. 20 most important features illustrating SHAP feature importance values for Subset-IGD_FP on CSMLP model, focusing on "Sweet" and "Green" odor classes.

## Imbalance measures values for IGD and Subset-IGD Datasets

The manuscript discusses our observation of significant label imbalance in both the IGD and Subset-IGD datasets. To mitigate this, we used two resampling techniques: ML-RUS and ML-ROS. Table 2 shows the effect of resampling on various imbalance measures. The table shows that both CVIR and MeanIR have significantly reduced after the resampling of both datasets with ML-ROS and ML-RUS.

| **Method** | **Sampling %** | **IGD** | | | | | **Subset-IGD** | | | | |
|---|---|---|---|---|---|---|---|---|---|---|---|
| | | **Card** | **Dens** | **CVIR** | **MaxIR** | **MeanIR** | **Card** | **Dens** | **CVIR** | **MaxIR** | **MeanIR** |
| **Resampled using ML-RUS** | **20** | 3.121 | 0.029 | 1.495 | 276 | 30.788 | 3.219 | 0.030 | 2.071 | 589 | 42.956 |
| | **30** | 3.118 | 0.028 | 1.434 | 296.6 | 31.431 | 3.172 | 0.029 | 1.806 | 505 | 36.082 |
| **Resampled using ML-ROS** | **20** | 3.102 | 0.028 | 0.791 | 83.136 | 20.423 | 3.168 | 0.029 | 2.279 | 631 | 27.355 |
| | **30** | 3.087 | 0.028 | 0.695 | 63.069 | 0.322 | 3.178 | 0.029 | 2.471 | 631 | 24.886 |

**Table 2.** Table shows values of imbalance measures for IGD and Subset-IGD Datasets for resampled data using ML-RUS and ML-ROS at varying percentages.

## References


1. Lin TY, Goyal P, Girshick R, He K, Dollar P. Focal Loss for Dense Object Detection. IEEE Transactions on Pattern Analysis and Machine Intelligence. 2020;42(2):318–327. doi:10.1109/tpami.2018.2858826.

2. Wang YX, Ramanan D, Hebert M. Learning to Model the Tail. In: Guyon I, Luxburg UV, Bengio S, Wallach H, Fergus R, Vishwanathan S, et al., editors. Advances in Neural Information Processing Systems. vol. 30. Curran Associates, Inc.; 2017.Available from: https://proceedings.neurips.cc/paper_files/paper/2017/file/147ebe637038ca50a1265abac8dea181-Paper.pdf.

3. Mikolov T, Sutskever I, Chen K, Corrado G, Dean J. Distributed Representations of Words and Phrases and their Compositionality; 2013. Available from: https://arxiv.org/abs/1310.4546.

4. Huang C, Li Y, Loy CC, Tang X. Learning Deep Representation for Imbalanced Classification. In: 2016 IEEE Conference on Computer Vision and Pattern Recognition (CVPR); 2016. p. 5375–5384.

5. Keras Developers. Keras Documentation - Optimizers; 2023. Available from: https://keras.io/api/optimizers/.

6. Nohara Y, Matsumoto K, Soejima H, Nakashima N. Explanation of machine learning models using shapley additive explanation and application for real data in hospital. Computer Methods and Programs in Biomedicine. 2022;214:106584. doi:10.1016/j.cmpb.2021.106584.
